# The prevalence of osteoporosis in rheumatoid arthritis patient: a systematic review and meta-analysis

**DOI:** 10.1038/s41598-022-20016-x

**Published:** 2022-09-23

**Authors:** Samaneh Moshayedi, Baharak Tasorian, Amir Almasi-Hashiani

**Affiliations:** 1 Student Research Committee, Arak University of Medical Sciences, Arāk, Iran; 2 Department of Rheumatology, School of Medicine, Arak University of Medical Sciences, Arāk, Iran; 3 Department of Epidemiology, School of Health, Arak University of Medical Sciences, Golestan St., Arāk, Iran; 4 Traditional and Complementary Medicine Research Center, Arak University of Medical Sciences, Arāk, Iran

**Keywords:** Medical research, Rheumatology, Risk factors

## Abstract

Osteoporosis (OP) is one of the most commonly known extra-articular complications of rheumatoid arthritis (RA). Since the prevalence of OP is diverse in different studies and there is no general consensus about it, in this systematic review, we aimed to investigate the global prevalence of OP among RA patients. In this review, three databases including Medline via PubMed, Scopus, and Web of Science (Clarivate analytics) were searched by various keywords. After screening of retrieved papers, the related data of included papers were extracted and analyzed. To assess the risk of methodological bias of included studies, quality assessment checklist for prevalence studies was used. Because of heterogeneity among studies, random-effect model was used to pooled the results of primary studies. In this review, the results of 57 studies were summarized and the total included sample size was 227,812 cases of RA with 64,290 cases of OP. The summary point prevalence of OP among RA was estimated as 27.6% (95%CI 23.9–31.3%). Despite significant advances in prevention, treatment and diagnostic methods in these patients, it still seems that the prevalence of OP in these patients is high and requires better and more timely interventions.

## Introduction

Rheumatoid arthritis (RA) is one of the most common autoimmune diseases that in the early stages of the disease begins with pain and symmetrical swelling of the small joints of the hands, feet, swelling of the soft tissue around the joint and morning stiffness and fatigue^1–4^ and it is characterized by persistent synovitis and progressive destruction of symmetrical multi-joints and intra-articular manifestations including subchondral lesions, decreased bone mass, and reduced generalized bone density^4–7^. The prevalence of RA in the general population is about 1%, but is more common in the 50 s and 60 s and is higher in women than men^8,9^.

Osteoporosis (OP) is one of the most known common extra-articular complications of RA^10^ and its prevalence in RA patients is almost twice that of the general population^4,11,12^. OP is a systemic skeletal disease characterized by decreased bone mineral density and its complication (increased fragility and fracture due to reduced resistance to torsion and compression)^7,13^. Bone fragility in people with RA includes a combination of systemic inflammation, circulating autoantibodies, and proinflammatory cytokines (IL1, IL6, TNF, etc.)^11,14^. Chronic inflammation in people with RA affects bone metabolism and disrupts the normal resorption cycle and reduces localized and generalized bone mineral density (BMD)^15^.

Decreased bone mass can also be affected by factors such as disease severity, gender, especially after menopause, decreased vitamin D levels, advanced age, using corticosteroids and disease-modifying anti-rheumatic drugs (DMRADs) and decreased mobility^12,16^. In the US, data show that osteoporotic fractures account for about one-third of RA-related mortality^5^. Fractures increase morbidity and mortality, reduce quality of life, reduce independent functioning of people, especially in old age, and increase economic burden^6,17^. Vertebral fracture is one of the most common fractures due to decreased BMD, which causes limitation of activity, disability, kyphosis and decreased pulmonary function^10,18,19^.

The diagnosis of OP is made by measuring bone marrow density by dual x ray absorptiometry of the lumbar vertebrae, which according to World Health Organization (WHO) classification: T > − 1 is normal, − 1 > T > − 2.5 is osteopenia and T < − 2.5 is OP^20^.

Despite advances in the identification of the destructive mechanism and pharmacological treatment of RA, the complications associated with this disease are still common. So, screening and assessing the prevalence of OP and proper management, especially in relation to timely identification, is essential to prevent fractures. For this reason, in this study, we systematically reviewed the international databases and the results of related papers were pooled regarding the prevalence of OP.

## Methods

### Study design

This is a systematic review and meta-analysis study. In this study, three international databases were systematically searched using different keywords. The “Preferred Reporting Items for Systematic Reviews and Meta-Analyses (PRISMA)”^21^ and “Cochrane Handbook for Systematic Reviews of Interventions”^22^ were used to report the results.

### Search strategy

To find related articles, a combination of related keywords was used in three databases including Medline via PubMed, Scopus, and Web of Science (Clarivate analytics). The keywords used included a combination of the suggested words by Medical Subject Heading (MeSH) and other related words. The search query used in PubMed was as follows: ((("Arthritis, Rheumatoid"[Mesh] OR "Rheumatoid Arthritis"[tw] OR "Rheumatoid"[tw]) AND ("Osteoporosis"[Mesh] OR "Osteoporosis"[tw] OR "Osteoporo*"[tw] OR " Bone Loss"[tw] OR "Osteopenia"[tw] OR "Bone Density"[Mesh] OR "Bone Density"[tw] OR "Bone Mineral Density"[tw])) AND ("Prevalence"[Mesh] OR "Incidence"[tw] OR "Epidemiology"[Mesh] OR "epidemiology" [Subheading] OR "Incidence"[Mesh] OR "Incidence"[tw])) NOT ("Clinical Trial" [Publication Type] OR "Controlled Clinical Trial" [Publication Type] OR "Clinical Trial, Phase III" [Publication Type]). Finally, the search filtered to human studies and English language studies. The adapted keywords were used to search in Scopus and Web of Science databases. The detailed search strategy was presented in Box 1. Databases were searched by two authors (AAH and SM) on June 22, 2021, and to find gray literatures, Google Scholar, and references of remaining articles manually searched.

**Box 1:** The search strategy in PubMed.

Search	Query	Results
#5	Search: ((("Arthritis, Rheumatoid"[Mesh] OR "Rheumatoid Arthritis"[tw] OR "Rheumatoid"[tw]) AND ("Osteoporosis"[Mesh] OR "Osteoporosis"[tw] OR "Osteoporo*"[tw] OR " Bone Loss"[tw] OR "Osteopenia"[tw] OR "Bone Density"[Mesh] OR "Bone Density"[tw] OR "Bone Mineral Density"[tw])) AND ("Prevalence"[Mesh] OR "Incidence"[tw] OR "Epidemiology"[Mesh] OR "epidemiology" [Subheading] OR "Incidence"[Mesh] OR "Incidence"[tw])) NOT ("Clinical Trial" [Publication Type] OR "Controlled Clinical Trial" [Publication Type] OR "Clinical Trial, Phase III" [Publication Type]) Filters: Humans, English Sort by: Most Recent	527
#4	Search: "Clinical Trial" [Publication Type] OR "Controlled Clinical Trial" [Publication Type] OR "Clinical Trial, Phase III" [Publication Type] Sort by: Most Recent	897,690
#3	Search: "Prevalence"[Mesh] OR "Incidence"[tw] OR "Epidemiology"[Mesh] OR "epidemiology" [Subheading] OR "Incidence"[Mesh] OR "Incidence"[tw] Sort by: Most Recent	2,895,709
#2	Search: "Osteoporosis"[Mesh] OR "Osteoporosis"[tw] OR "Osteoporo*"[tw] OR " Bone Loss"[tw] OR "Osteopenia"[tw] OR "Bone Density"[Mesh] OR "Bone Density"[tw] OR "Bone Mineral Density"[tw] Sort by: Most Recent	166,724
#1	Search: "Arthritis, Rheumatoid"[Mesh] OR "Rheumatoid Arthritis"[tw] OR "Rheumatoid"[tw] Sort by: Most Recent	162,057

### Study selection and screening

To find and screen related articles, all retrieved articles were entered into Endnote software, and duplicate articles were first identified and removed. Then, in the next step, the articles were screened in terms of title and abstract, and the irrelevant articles were deleted. In the next step, the full text of the related articles was screened, and the articles that met the inclusion criteria and related data were studied and the required information was extracted from them. All these steps were performed by two authors (SM and AAH) independently and in case of disagreement between the two authors, a decision was made after consultation.

### Inclusion and exclusion criteria

Articles with English full-text that were indexed in desired databases up to June 22, 2021 (from 1962 to 2021) were searched and there was no publication time limit. All observational studies in which the prevalence of OP has been reported in patients with RA have been included in the study. All clinical trials, letter to editor, editorials, review articles, commentaries, case reports, case series studies and papers with no relevant data were excluded.

### Data extraction

The required data were extracted from the articles by two authors (SM and AAH) and in case of disagreement, the final decision was made after consultation. The extracted data were entered into a designed checklist in Excel software. This data includes first author’s name, year of publication, duration of patient’s recruitment, mean age, mean of disease duration, countries, the score of risk of bias, sample size, number of cases with OP and prevalence of OP.

### Risk of bias

To assess the risk of bias of included studies, quality assessment checklist for prevalence studies which was developed by Hoy et al.^23^ was used. This checklist consists of nine items, each item has a score of 0 or 1. The score of 0 indicates the low risk and score of 1 indicates the high risk. The total score of checklists ranges from 0 to 9, which categorized in three levels; 0–3, 4–6 and 7–9 as low, moderate and high risk, respectively.

### Statistical analysis

The I^2^ statistic with as well as chi-square test was used to assess the heterogeneity across the included studies. The results revealed that there was noteworthy heterogeneity between studies, and a meta-regression to find the source of heterogeneity and a subgroup analysis were done, and because of heterogeneity, the random-effect model was used to pooled the extracted prevalence with “metaprop” command^24^. Egger’s linear regression and funnel plot were used to explore the publication bias and trim and fill method was used to estimate the prevalence in case of publication bias. To recognize the effect of each study on the pooled prevalence, a sensitivity analysis was conducted. All analyses were conducted using Stata software version 13 (Stata Corp, College Station, TX, USA).

### Ethics approval and consent to participate

This study was approved by Ethical Committee of Arak University of Medical Sciences (Code: IR.ARAKMU.REC.1399.259).

## Result

### Study selection and study characteristics

**Figure 1:**
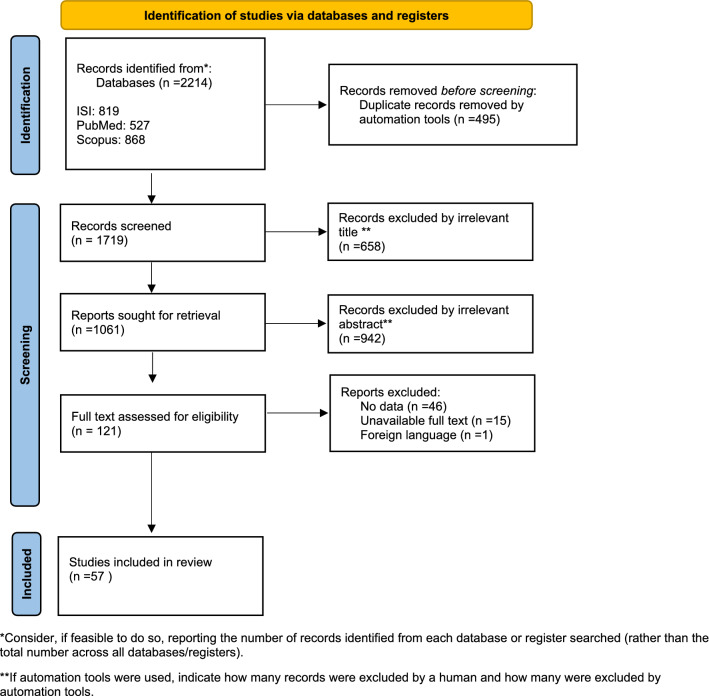
Flow diagram of the literature search for studies included in meta-analysis.

The process of study selection is presented in the PRISMA flow diagram^25^ (Fig. 1). First, after searching the desired databases, we retrieved 2214 primary studies (PubMed/Medline: 527, Scopus: 868, and Web of Science: 819). Then, 495 articles were removed due to duplication and 1719 studies were screened by title and abstract. Next, 658 papers were excluded by irrelevant title and 942 papers were excluded by irrelevant abstract. After that, the full text of 121 remained papers were assessed for eligibility and 62 papers were excluded (no data: 46 papers, unavailable full text: 15 papers and foreign language: 1 paper). Finally, data from 57 articles^1,3,4,7,8,11,13,16,18,20,26–72^ were entered into the meta-analysis.

The sample size of imported articles ranged from 37 to 142,955. The oldest article was in 1962 and the most recent article was in 2021, and the reported prevalence of OP among RA patients varied from 3.7% to 62.2%. Further details regarding the selected studies are described in Table 1.

**Table 1:** Characteristics of the primary studies included in the meta-analysis.

Id	Author	Year	Countries	Prevalence	Sample size	Mean age	Disease duration	Risk score	References
1	Venter G	2021	Australia	14.7	109	59.5	204	Moderate	^69^
2	Tavassoli S	2021	Iran	8.5	129	56.33	83	Low	^7^
3	Pierini F. S	2021	Argentina	36.5	74	62.1	114	Low	^13^
4	Hu Z	2021	China	54.7	340	59.4	66	Low	^4^
5	Tong J	2019	China	33.6	865	55.6	113	Low	^68^
6	Lindner L	2020	Germany	6	5423	63	168	Low	^51^
7	Hu Z	2020	China	62.1	452	58	67	Low	^43^
8	Yan S	2019	China	4.19	788	56	48	Low	^72^
9	Wafa H	2018	Tunisia	48	173	54.1	98	Low	^71^
10	Tong H	2018	China	35	320	54.1	72	Low	^67^
11	Luque Ramos A	2019	Germany	25.9	2535	62.5		Moderate	^52^
12	Fauny M	2019	France	26.7	105	61.1	144	Low	^30^
13	Phuan-udom R	2018	Thailand	5	232	61.1	155	Moderate	^11^
14	Panopoulos S	2018	Greece	21.4	182	61.6	108	Low	^62^
15	Mohd-Tahir N. A	2017	Malaysia	29	93	61.7	66	Moderate	^59^
16	Kweon S. M	2018	South Korea	19.7	76	64.5	37.5	Low	^47^
17	Kim D	2018	South Korea	33.8	142,955	54.2	24.5	High	^45^
18	Heidari B	2018	Iran	30.8	39	50.6	108	Low	^41^
19	Gabdulina G	2018	Kazakhstan	45.1	406	50.6	61.6	Low	^31^
20	Ene C. G	2018	Romania	32.2	62	49.43		Low	^29^
21	Choi S. T	2018	South Korea	33.4	479	61.5	53	Low	^27^
22	Rossini M	2017	Italian	35	183	64	108	Moderate	^65^
23	Meng J	2017	China	41.07	168	54.3	146.5	Low	^20^
24	Makhdoom A	2017	Pakistan	40.6	229	46.4		Low	^53^
25	Galarza-Delgado D. A	2017	Mexico	19.1	225	55.7	114	Moderate	^32^
26	Singh S	2016	India	5.9	51	45		Low	^1^
27	Lee J. H	2016	South Korea	46.8	1322	63.7	145	Low	^50^
28	Kim D	2016	South Korea	5.5	5376	58.8	117.5	Low	^44^
29	Innala L	2016	Sweden	3.7	726	55.6	80.5	Moderate	^3^
30	Garip Y	2016	Turkey	21.2	160	53.6	145	Low	^33^
31	Bautista-Molano W	2016	Colombia	17.3	1652	58	110.5	Low	^26^
32	Piao H. H	2015	China	21.6	37	64.4		Moderate	^63^
33	Mohammad A	2013	Ireland	59	603	57	180	Low	^58^
34	Lee J. H	2014	South Korea	59.1	545	57	135	Low	^49^
35	Lee J. H	2014	South Korea	51	100	61.2	78	Low	^48^
36	Hauser B	2014	UK	29.9	304	63.5	115	Low	^40^
37	Gron K. L	2014	34 countries	17.6	9874	54.9	97	Moderate	^37^
38	Mobini m	2012	Iran	32.3	121	55.7	121	Low	^57^
39	Lee S. G	2012	South Korea	22.1	299	52.4	32	Low	^16^
40	Gonzalez-Lopez L	2012	Mexico	24.1	191	52	132	Low	^36^
41	Ghazi M	2012	France	55.4	101	56.1	179.5	Low	^34^
42	Vis M	2011	(Norway, UK, Netherlands)	35	102	61	204	Moderate	^70^
43	Dao H. H	2011	Vietnam	27.6	105	56.3	21	Low	^28^
44	Kim S. Y	2010	USA	18	47,034	55		Low	^46^
45	El Maghraoui A	2010	Morocco	44.2	172	49.4	101	Low	^18^
46	Shankar S	2008	India	22	84	33.9	60	Low	^66^
47	Sarkis K. S	2009	Brazil	25.3	83	55	92.5	Low	^8^
48	Richards J. S	2009	USA	18	282	65.4	156	Moderate	^64^
49	Oelzner P	2008	Germany	47.8	551	58.4	144	Low	^61^
50	Haugen I. K	2007	Norway	19.4	194	60.9		Low	^39^
51	Nolla J. M	2006	Spain	13	187	60.34	109	Low	^60^
52	Mikuls T. R	2005	USA	4.7	175	60	109	Low	^56^
53	Heidari B	2004	Iran	25	88	52.6	84	Low	^42^
54	Manrique F	2003	Venezuela	29.4	85	45.3	113	Low	^54^
55	Haugeberg G	2000	Norway	4.2	394	54.8	156	Moderate	^38^
56	Gilboe I. M	2000	Norway	5	75	45	95	Low	^35^
57	Moconkey B	1962		30.3	97	63.1	14.7	Moderate	^55^

### Risk of bias within studies

The risk of bias of included studies was assessed by the quality assessment checklist for prevalence studies. The results showed that the risk of bias of 75.4% (n = 43), 22.8% (n = 13) and 1.75% (n = 1) of included papers were low, moderate and high, respectively.

### Quantitative data synthesis

**Figure 2:**
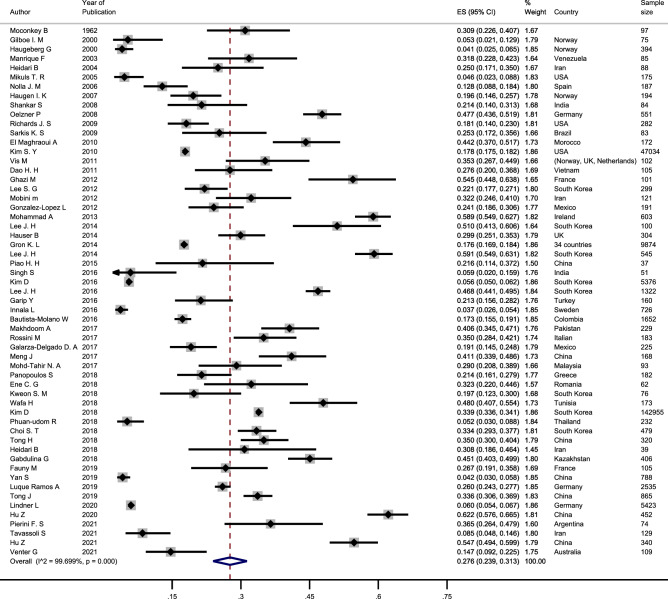
Forest plot showing the prevalence of osteoporosis among rheumatoid arthritis patient.

In this review, the results of 57 studies were summarized and the total included sample size was 227,812 cases of RA with 64,290 cases of OP. Due to the significant heterogeneity across studies, the random-effect model was used to pool the reported prevalence. The summary point prevalence was estimated as 27.6% (95%CI: 23.9–31.3%) (Table 2; Fig. 2).

**Table 2:** Summary of meta-analysis results and subgroups analysis.

Groups	No of studies	Prevalence rate		Heterogeneity		
		ES (95%CI)	Model	Chi square	P value	I square (%)
**Date of publication**						
1962–2010	14	21.6% (15.8–27.4)	Random	553.1	0.001	97.6%
2011–2015	12	36.2% (24.5–47.8)	Random	875.9	0.001	98.7%
2016–2021	31	27.1% (20.7–33.4)	Random	15,203.5	0.001	99.8%
**Study risk score**						
Low risk	43	29.8% (26.2–33.5)	Random	5504.0	0.001	99.2%
Moderate	13	19.3% (13.9–24.7)	Random	705.8	0.001	98.3%
High risk	1	33.9% (33.6–34.1)	Random	–	–	–
**Continents**						
Asia	26	30.6% (23.2–38.0)	Random	9508.0	0.001	99.7%
Europe	17	25.6% (18.7–32.4)	Random	1803.9	0.001	99.1%
America	9	19.5% (15.9–23.1)	Random	96.1	0.001	91.6%
Africa	2	46.1% (40.8–51.3)	Random	–	–	–
**Overall**	57	27.6% (23.9–31.3)	Random	18,613.03	0.001	99.69%

### Heterogeneity and meta-regression

The obtained results revealed a significant heterogeneity across primary included studies (heterogeneity chi-square = 18587.5, d.f = 56, p = 0.001, I-square (variation in prevalence attributable to heterogeneity) = 99.7%, estimate of between-study variance Tau-square = 0.019), for this reason, random-effect model was used to pool the reported prevalence. In addition, meta-regression method was used to find the heterogeneity source, and in meta-regression, we included sample size, study reign (continents), date of publication and risk score of studies and in the meta-regression model, none of these variables were significant. Finally, in addition to using a random effect model, subgroup analysis was performed based on study reigns (continents), date of publication and risk score of studies.

### Sub-group analysis

As it was showed in Table 2, according to the subgroup analysis based on the data of publication, the highest prevalence was in studies conducted during 2011–2015 (36.2% (95%CI 24.5–47.8)), followed by 2016–2021 (27.1% (95%CI 20.7–33.4)) and before 2010 (21.6% (95%CI 15.8–27.4)). The prevalence in studies with low and moderate risk score was 29.8% (95%CI 26.2–33.5) and 36.2% (95%CI 24.5–47.8), respectively. Based on the study reign, the highest prevalence of OP was in Africa (46.1% (95%CI 40.8–51.3)), followed by Asia (30.6% (95%CI 23.2–38.0)), Europe (25.6% (95%CI 18.7–32.4)), and the Americas (19.5% (95%CI 15.9–23.1)).

### Risk of bias across studies

**Figure 3:**
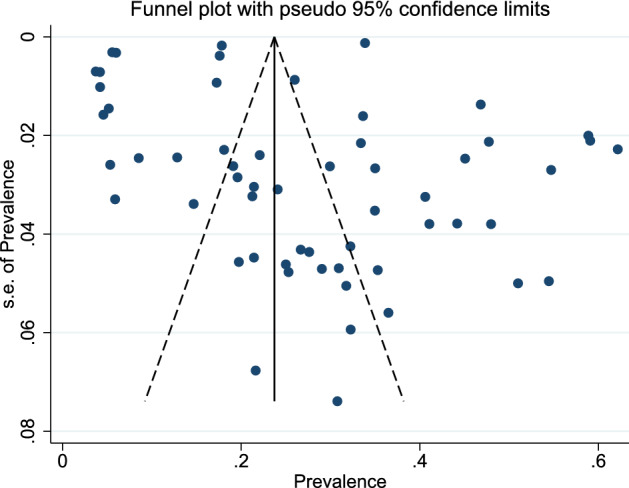
Funnel plot to check the publication bias.

Egger's test for small-study effects was performed to check for possibility of publication bias. The obtained results of Egger's test (z = 2.13, p = 0.033) suggested that there is an evidence of publication bias. In addition to Egger's test, the asymmetry in the funnel plot (Fig. 3) emphasized the existence of publication bias. For this reason, trim and fill method was used to estimate the OP prevalence and, the prevalence was estimated to be 23.3% (95%CI 19.7–26.8%) using random-effect model.

### Sensitivity analysis

To investigate the effect of each study on the pooled prevalence, we conducted a sensitivity analysis in which pooled prevalence are estimated omitting one study at a time. The highest pooled prevalence (28.1%, 95%CI 24.4–31.8%) was obtained by omitting the study of Innala et al.^3^ and the lowest pooled prevalence (27.0%, 95%CI 23.3–30.7%) was obtained by omitting the study of Hu et al.^43^.

## Discussion

In this study, 57 primary studies with a total population of 227,812 cases were included in the meta-analysis, and according to the obtained results, OP prevalence among RA patients is 27.6%. The subgroup analysis based on the data of publication suggested that the highest prevalence was found in studies conducted during 2011–2015 (36.2%), followed by 2016–2021 (27.1%). The prevalence in studies with low and moderate risk score was 29.8% and 36.2%, respectively. Based on the study region, the highest prevalence of OP was in Africa (46.1%), followed by Asia (30.6%), Europe (25.6%), and the Americas (19.5%).

RA is a chronic inflammatory disease that, it leads to localized and generalized reduction in bone density and eventually causes OP^73^. Bone fractures are one of the most common complications in RA patients caused by OP and is associated with poor prognosis in old age and low quality of life^74^. According to the results, the prevalence of OP varies in different countries and continents, which can be attributed to the population density and different time of studies, age, economic situation and lack of government attention to the issue. In addition, difference in the quality of providing medical services, access to osteoporosis screening methods, and controlling the risk factors related to it and also preventing the disease play an important role.

A systematic review conducted by Salari et al.^75^ in 2021 to estimate the prevalence of OP in the general population. After review of 86 included studies, the worldwide prevalence of OP is estimated as 18.3% and in Asia, Europe, the Americas and Africa it was estimated as 16.7, 18.6, 12.4, and 39.5%, respectively. According to their study, the estimated prevalence was lower compared to our study, the reason is that people with RA have a higher risk of developing OP than the general population. In our study, similar to the study of Salari et al., the prevalence was lower in the Americas and higher in Africa followed by Asian and European countries.

In a meta-analysis, Ramírez et al.^76^ reviewed the results of 45 articles and found that the prevalence of OP in patients with axial spondylarthritis varies from 11.7 to 34.4%. In another meta-analysis study conducted on the general Chinese population, Chen et al. revealed that the prevalence of OP ranged from 1 to 85%^77^. The results of previous studies^78^ have shown that the prevalence of OP in people with RA is about 30%. The findings of our study had a similar estimate.

The results of our study and previous studies have shown that the prevalence of OP in people with RA is higher than the general population. Various factors play a role in increasing the prevalence of OP in patients with rheumatoid arthritis, the most important of which are continuous inflammation, glucocorticoid use, reduced physical activity due to old age and disability, and the use of DMARDs^78^.

In this study we investigated the results of 227,812 cases of RA with 64,290 cases of OP and it should be highlighted that 142,955 of these cases (63%) are related to the study conducted by Kim D et al.^45^ in South Korea, and the prevalence of OP reported as 33.8% in their study.

The incidence of OP is caused by several factors among RA patients. In the pathogenesis of inflammation and reduction of BMD, various factors in immune system, are involved such as hyper-expression and the effect of autoantibodies against citrullinated proteins, pro-inflammatory cytokine secretion, and receptor activator of NF-kappa B ligand derived from T-cell^79^. Immunosuppressive drugs such as glucocorticoids and DMARDs are used to treat RA. Glucocorticoids with their anti-inflammatory effects can prevent local and systemic decrease in BMD. Furthermore, DMARDs are used to achieve remission, and evidence suggests that DMARDs prevent structural damage to cartilage and bone^80,81^.

Decreased vitamin D intake is associated with an increased risk of RA, and also, vitamin D deficiency is associated with disease activity in patients with RA^82^. Therefore, vitamin D deficiency can be one of the common causes of RA and OP. The results of a meta-analysis study showed that vitamin D deficiency in RA patients is significantly higher than healthy individuals and serum vitamin D levels are inversely related with disease activity^83^.

The results suggest that the prevalence of RA has been declining in recent years, which may be attributed to the increase of human knowledge about drugs that suppress RA and timely imaging studies for early diagnosis and adequate treatment. Among the four continents (i.e., Africa, Asia, the Americas and Europe), Asia has the most prevalent of OP followed by Europe. In most studies, due to the higher risk of women with RA, the majority of the population was women and most of them were in menopausal ages and is associated with estrogen reduction, which is an important risk factor to increase prevalence of OP. It should be noted that because most studies used the DEXA method to evaluate OP, there is lower error in the diagnostic method. Although in some countries, limited studies have been conducted, but it can be said that the prevalence of OP in RA is high and it is necessary to have a decent platform for screening and timely use of medications and patients’ education to reduce modifiable risk factors to reduce the incidence of OP to minimize the complications.

One of the main limitations of the study is the lack of sufficient number of studies conducted in each area (for example only two studies from the African continent were included in this meta-analysis), which makes it difficult to generalize the results. Also, in other WHO regions, studies have been conducted in limited countries, which makes it impossible to show the true prevalence in each region. On the other hand, in a number of studies in which people were treated with corticosteroids and DMARDs, the rate of bone mass reduction was not examined separately, so it was not possible to compare between drug users and other people. Finally, due to the disparity of results in different continents and countries, more comprehensive studies are recommended to make a better conclusion.

## Conclusion

Despite significant advances in prevention, treatment and diagnostic methods in RA patients, it still seems that the prevalence of OP in these patients is high and requires better and timelier interventions.

## Supplementary Information

Supplementary Information.

## Data Availability

All data for the analyses is available from the corresponding author on request.

## Abbreviations

PRISMA: Preferred reporting items for systematic reviews and meta-analyses
OP: Osteoporosis
RA: Rheumatoid arthritis
CI: Confidence interval
DMARDs: Disease-modifying anti-rheumatic drugs
MeSH: Medical subject headings
BMD: Bone mineral density
WHO: World Health Organization
