# Supplementary material for: The prevalence of osteoporosis in rheumatoid arthritis patient: a systematic review and meta-analysis
Source: Sci Rep. 2022 Sep 23;12:15844. doi: 10.1038/s41598-022-20016-x (PMC9508181; doi:10.1038/s41598-022-20016-x)
Supplement: Supplementary file 1 — Supplementary Information. [file 41598_2022_20016_MOESM1_ESM.docx]

**Keywords:**

"Arthritis, Rheumatoid"[Mesh] OR "Rheumatoid Arthritis"[tw] OR "Rheumatoid"[tw]

**AND**

"Osteoporosis"[Mesh] OR "Osteoporosis"[tw] OR "Osteoporo*"[tw] OR " Bone Loss"[tw] OR “Osteopenia"[tw] OR "Bone Density"[Mesh] OR "Bone Density"[tw] OR "Bone Mineral Density"[tw]

**AND**

"Prevalence"[Mesh] OR "Incidence"[tw] OR "Epidemiology"[Mesh] OR “epidemiology" [Subheading] OR "Incidence"[Mesh] OR "Incidence"[tw]

**NOT**

"Clinical Trial" [Publication Type] OR "Controlled Clinical Trial" [Publication Type] OR "Clinical Trial, Phase III" [Publication Type]

History and Search Details – **PubMed (on June 22, 2021)**

| Search | Query | Results |
| --- | --- | --- |
| #7 | Search: ((("Arthritis, Rheumatoid"[Mesh] OR "Rheumatoid Arthritis"[tw] OR "Rheumatoid"[tw]) AND ("Osteoporosis"[Mesh] OR "Osteoporosis"[tw] OR "Osteoporo*"[tw] OR " Bone Loss"[tw] OR "Osteopenia"[tw] OR "Bone Density"[Mesh] OR "Bone Density"[tw] OR "Bone Mineral Density"[tw])) AND ("Prevalence"[Mesh] OR "Incidence"[tw] OR "Epidemiology"[Mesh] OR "epidemiology" [Subheading] OR "Incidence"[Mesh] OR "Incidence"[tw])) NOT ("Clinical Trial" [Publication Type] OR "Controlled Clinical Trial" [Publication Type] OR "Clinical Trial, Phase III" [Publication Type]) Filters: Humans, English Sort by: Most Recent | [527](https://pubmed.ncbi.nlm.nih.gov/?term=%28%28%28%22Arthritis%2C+Rheumatoid%22%5BMesh%5D+OR+%22Rheumatoid+Arthritis%22%5Btw%5D+OR+%22Rheumatoid%22%5Btw%5D%29+AND+%28%22Osteoporosis%22%5BMesh%5D+OR+%22Osteoporosis%22%5Btw%5D+OR+%22Osteoporo%2A%22%5Btw%5D+OR+%22+Bone+Loss%22%5Btw%5D+OR+%22Osteopenia%22%5Btw%5D+OR+%22Bone+Density%22%5BMesh%5D+OR+%22Bone+Density%22%5Btw%5D+OR+%22Bone+Mineral+Density%22%5Btw%5D%29%29+AND+%28%22Prevalence%22%5BMesh%5D+OR+%22Incidence%22%5Btw%5D+OR+%22Epidemiology%22%5BMesh%5D+OR+%22epidemiology%22+%5BSubheading%5D+OR+%22Incidence%22%5BMesh%5D+OR+%22Incidence%22%5Btw%5D%29%29+NOT+%28%22Clinical+Trial%22+%5BPublication+Type%5D+OR+%22Controlled+Clinical+Trial%22+%5BPublication+Type%5D+OR+%22Clinical+Trial%2C+Phase+III%22+%5BPublication+Type%5D%29&filter=hum_ani.humans&filter=lang.english&sort=date&size=20) |
| #6 | Search: ((("Arthritis, Rheumatoid"[Mesh] OR "Rheumatoid Arthritis"[tw] OR "Rheumatoid"[tw]) AND ("Osteoporosis"[Mesh] OR "Osteoporosis"[tw] OR "Osteoporo*"[tw] OR " Bone Loss"[tw] OR "Osteopenia"[tw] OR "Bone Density"[Mesh] OR "Bone Density"[tw] OR "Bone Mineral Density"[tw])) AND ("Prevalence"[Mesh] OR "Incidence"[tw] OR "Epidemiology"[Mesh] OR "epidemiology" [Subheading] OR "Incidence"[Mesh] OR "Incidence"[tw])) NOT ("Clinical Trial" [Publication Type] OR "Controlled Clinical Trial" [Publication Type] OR "Clinical Trial, Phase III" [Publication Type]) Filters: Humans Sort by: Most Recent | [608](https://pubmed.ncbi.nlm.nih.gov/?term=%28%28%28%22Arthritis%2C+Rheumatoid%22%5BMesh%5D+OR+%22Rheumatoid+Arthritis%22%5Btw%5D+OR+%22Rheumatoid%22%5Btw%5D%29+AND+%28%22Osteoporosis%22%5BMesh%5D+OR+%22Osteoporosis%22%5Btw%5D+OR+%22Osteoporo%2A%22%5Btw%5D+OR+%22+Bone+Loss%22%5Btw%5D+OR+%22Osteopenia%22%5Btw%5D+OR+%22Bone+Density%22%5BMesh%5D+OR+%22Bone+Density%22%5Btw%5D+OR+%22Bone+Mineral+Density%22%5Btw%5D%29%29+AND+%28%22Prevalence%22%5BMesh%5D+OR+%22Incidence%22%5Btw%5D+OR+%22Epidemiology%22%5BMesh%5D+OR+%22epidemiology%22+%5BSubheading%5D+OR+%22Incidence%22%5BMesh%5D+OR+%22Incidence%22%5Btw%5D%29%29+NOT+%28%22Clinical+Trial%22+%5BPublication+Type%5D+OR+%22Controlled+Clinical+Trial%22+%5BPublication+Type%5D+OR+%22Clinical+Trial%2C+Phase+III%22+%5BPublication+Type%5D%29&filter=hum_ani.humans&sort=date&size=20) |
| #5 | Search: ((("Arthritis, Rheumatoid"[Mesh] OR "Rheumatoid Arthritis"[tw] OR "Rheumatoid"[tw]) AND ("Osteoporosis"[Mesh] OR "Osteoporosis"[tw] OR "Osteoporo*"[tw] OR " Bone Loss"[tw] OR "Osteopenia"[tw] OR "Bone Density"[Mesh] OR "Bone Density"[tw] OR "Bone Mineral Density"[tw])) AND ("Prevalence"[Mesh] OR "Incidence"[tw] OR "Epidemiology"[Mesh] OR "epidemiology" [Subheading] OR "Incidence"[Mesh] OR "Incidence"[tw])) NOT ("Clinical Trial" [Publication Type] OR "Controlled Clinical Trial" [Publication Type] OR "Clinical Trial, Phase III" [Publication Type]) Sort by: Most Recent | [652](https://pubmed.ncbi.nlm.nih.gov/?term=%28%28%28%22Arthritis%2C+Rheumatoid%22%5BMesh%5D+OR+%22Rheumatoid+Arthritis%22%5Btw%5D+OR+%22Rheumatoid%22%5Btw%5D%29+AND+%28%22Osteoporosis%22%5BMesh%5D+OR+%22Osteoporosis%22%5Btw%5D+OR+%22Osteoporo%2A%22%5Btw%5D+OR+%22+Bone+Loss%22%5Btw%5D+OR+%22Osteopenia%22%5Btw%5D+OR+%22Bone+Density%22%5BMesh%5D+OR+%22Bone+Density%22%5Btw%5D+OR+%22Bone+Mineral+Density%22%5Btw%5D%29%29+AND+%28%22Prevalence%22%5BMesh%5D+OR+%22Incidence%22%5Btw%5D+OR+%22Epidemiology%22%5BMesh%5D+OR+%22epidemiology%22+%5BSubheading%5D+OR+%22Incidence%22%5BMesh%5D+OR+%22Incidence%22%5Btw%5D%29%29+NOT+%28%22Clinical+Trial%22+%5BPublication+Type%5D+OR+%22Controlled+Clinical+Trial%22+%5BPublication+Type%5D+OR+%22Clinical+Trial%2C+Phase+III%22+%5BPublication+Type%5D%29&sort=date&size=20) |
| #4 | Search: "Clinical Trial" [Publication Type] OR "Controlled Clinical Trial" [Publication Type] OR "Clinical Trial, Phase III" [Publication Type] Sort by: Most Recent | [897,690](https://pubmed.ncbi.nlm.nih.gov/?term=%22Clinical+Trial%22+%5BPublication+Type%5D+OR+%22Controlled+Clinical+Trial%22+%5BPublication+Type%5D+OR+%22Clinical+Trial%2C+Phase+III%22+%5BPublication+Type%5D&sort=date&size=20) |
| #3 | Search: "Prevalence"[Mesh] OR "Incidence"[tw] OR "Epidemiology"[Mesh] OR "epidemiology" [Subheading] OR "Incidence"[Mesh] OR "Incidence"[tw] Sort by: Most Recent | [2,895,709](https://pubmed.ncbi.nlm.nih.gov/?term=%22Prevalence%22%5BMesh%5D+OR+%22Incidence%22%5Btw%5D+OR+%22Epidemiology%22%5BMesh%5D+OR+%E2%80%9Cepidemiology%22+%5BSubheading%5D+OR+%22Incidence%22%5BMesh%5D+OR+%22Incidence%22%5Btw%5D&sort=date&size=20) |
| #2 | Search: "Osteoporosis"[Mesh] OR "Osteoporosis"[tw] OR "Osteoporo*"[tw] OR " Bone Loss"[tw] OR "Osteopenia"[tw] OR "Bone Density"[Mesh] OR "Bone Density"[tw] OR "Bone Mineral Density"[tw] Sort by: Most Recent | [166,724](https://pubmed.ncbi.nlm.nih.gov/?term=%22Osteoporosis%22%5BMesh%5D+OR+%22Osteoporosis%22%5Btw%5D+OR+%22Osteoporo%2A%22%5Btw%5D+OR+%22+Bone+Loss%22%5Btw%5D+OR+%E2%80%9COsteopenia%22%5Btw%5D+OR+%22Bone+Density%22%5BMesh%5D+OR+%22Bone+Density%22%5Btw%5D+OR+%22Bone+Mineral+Density%22%5Btw%5D&sort=date&size=20) |
| #1 | Search: "Arthritis, Rheumatoid"[Mesh] OR "Rheumatoid Arthritis"[tw] OR "Rheumatoid"[tw] Sort by: Most Recent | [162,057](https://pubmed.ncbi.nlm.nih.gov/?term=%22Arthritis%2C+Rheumatoid%22%5BMesh%5D+OR+%22Rheumatoid+Arthritis%22%5Btw%5D+OR+%22Rheumatoid%22%5Btw%5D&sort=date&size=20) |

**Search History in ISI/WoS: (on June 22, 2021)**

| **Set** | **Results** | **Save History / Create AlertOpen Saved History** |
| --- | --- | --- |
| # 7 | [**819**](https://www1.wosgs.ir/summary.do?product=WOS&doc=1&qid=7&SID=E6tMc98H7BRXS6pu5cb&search_mode=AdvancedSearch&update_back2search_link_param=yes) | (#6) *AND***LANGUAGE:** (English) *AND* **DOCUMENT TYPES:** (Article)  *Indexes=SCI-EXPANDED, SSCI, A&HCI, CPCI-S, CPCI-SSH, BKCI-S, BKCI-SSH, ESCI Timespan=All years* |
| # 6 | [**1,028**](https://www1.wosgs.ir/summary.do?product=WOS&doc=1&qid=6&SID=E6tMc98H7BRXS6pu5cb&search_mode=AdvancedSearch&update_back2search_link_param=yes) | (#5 NOT #4) *AND***LANGUAGE:** (English)  *Indexes=SCI-EXPANDED, SSCI, A&HCI, CPCI-S, CPCI-SSH, BKCI-S, BKCI-SSH, ESCI Timespan=All years* |
| # 5 | [**1,103**](https://www1.wosgs.ir/summary.do?product=WOS&doc=1&qid=5&SID=E6tMc98H7BRXS6pu5cb&search_mode=CombineSearches&update_back2search_link_param=yes) | #3 AND #2 AND #1  *Indexes=SCI-EXPANDED, SSCI, A&HCI, CPCI-S, CPCI-SSH, BKCI-S, BKCI-SSH, ESCI Timespan=All years* |
| # 4 | [**205,381**](https://www1.wosgs.ir/summary.do?product=WOS&doc=1&qid=4&SID=E6tMc98H7BRXS6pu5cb&search_mode=AdvancedSearch&update_back2search_link_param=yes) | TS= ("Clinical Trial") OR TS= ("Controlled Clinical Trial")  *Indexes=SCI-EXPANDED, SSCI, A&HCI, CPCI-S, CPCI-SSH, BKCI-S, BKCI-SSH, ESCI Timespan=All years* |
| # 3 | [**4,205,126**](https://www1.wosgs.ir/summary.do?product=WOS&doc=1&qid=3&SID=E6tMc98H7BRXS6pu5cb&search_mode=AdvancedSearch&update_back2search_link_param=yes) | TS= ("Prevalence") OR TS= ("Incidence") OR TS=("Epidemiology") OR TS=(Frequency)  *Indexes=SCI-EXPANDED, SSCI, A&HCI, CPCI-S, CPCI-SSH, BKCI-S, BKCI-SSH, ESCI Timespan=All years* |
| # 2 | [**175,524**](https://www1.wosgs.ir/summary.do?product=WOS&doc=1&qid=2&SID=E6tMc98H7BRXS6pu5cb&search_mode=AdvancedSearch&update_back2search_link_param=yes) | TS= (Osteoporosis) OR TS= (Osteoporo*) OR TS= ("Bone Loss") OR TS=("Osteopenia") OR TS= ("Bone Density") OR TS= ("Bone Mineral Density")  *Indexes=SCI-EXPANDED, SSCI, A&HCI, CPCI-S, CPCI-SSH, BKCI-S, BKCI-SSH, ESCI Timespan=All years* |
| # 1 | [**184,910**](https://www1.wosgs.ir/summary.do?product=WOS&doc=1&qid=1&SID=E6tMc98H7BRXS6pu5cb&search_mode=GeneralSearch&update_back2search_link_param=yes) | **TOPIC:** ("Rheumatoid Arthritis") *OR* **TOPIC:** ("Rheumatoid")  *Indexes=SCI-EXPANDED, SSCI, A&HCI, CPCI-S, CPCI-SSH, BKCI-S, BKCI-SSH, ESCI Timespan=All years* |
|  |  |  |

## Search history-Scopus (on June 22, 2021)

| **#** | **Query** | **Results** |
| --- | --- | --- |
| 7 | ( ( ( TITLE-ABS-KEY ( "Arthritis, Rheumatoid" )  OR  TITLE-ABS-KEY ( "Rheumatoid Arthritis" )  OR  TITLE-ABS-KEY ( "Rheumatoid" ) ) )  AND  ( ( TITLE-ABS-KEY ( "Osteoporosis" )  OR  TITLE-ABS-KEY ( "Osteoporo*" )  OR  TITLE-ABS-KEY ( "Bone Loss" )  OR  TITLE-ABS-KEY ( "Osteopenia" )  OR  TITLE-ABS-KEY ( "Bone Density" )  OR  TITLE-ABS-KEY ( "Bone Mineral Density" ) ) )  AND  ( ( TITLE-ABS-KEY ( "Prevalence" )  OR  TITLE-ABS-KEY ( "Incidence" )  OR  TITLE-ABS-KEY ( "Epidemiology" )  OR  TITLE-ABS-KEY ( "Frequency" ) ) ) )  AND NOT  ( TITLE-ABS-KEY ( "Clinical Trial" ) )  AND  ( LIMIT-TO ( DOCTYPE ,  "ar" )  OR  LIMIT-TO ( DOCTYPE ,  "cp" ) )  AND  ( LIMIT-TO ( LANGUAGE ,  "English" ) )  AND  ( LIMIT-TO ( SRCTYPE ,  "j" ) ) View Less | [868 document results](https://www.scopus.com/search/history/results.uri?origin=searchhistory&shid=7) |
| 6 | ( ( ( TITLE-ABS-KEY ( "Arthritis, Rheumatoid" )  OR  TITLE-ABS-KEY ( "Rheumatoid Arthritis" )  OR  TITLE-ABS-KEY ( "Rheumatoid" ) ) )  AND  ( ( TITLE-ABS-KEY ( "Osteoporosis" )  OR  TITLE-ABS-KEY ( "Osteoporo*" )  OR  TITLE-ABS-KEY ( "Bone Loss" )  OR  TITLE-ABS-KEY ( "Osteopenia" )  OR  TITLE-ABS-KEY ( "Bone Density" )  OR  TITLE-ABS-KEY ( "Bone Mineral Density" ) ) )  AND  ( ( TITLE-ABS-KEY ( "Prevalence" )  OR  TITLE-ABS-KEY ( "Incidence" )  OR  TITLE-ABS-KEY ( "Epidemiology" )  OR  TITLE-ABS-KEY ( "Frequency" ) ) ) )  AND NOT  ( TITLE-ABS-KEY ( "Clinical Trial" ) ) View Less | [1,369 document results](https://www.scopus.com/search/history/results.uri?origin=searchhistory&shid=6) |
| 5 | TITLE-ABS-KEY ( "Clinical Trial" ) | [1,570,777 document results](https://www.scopus.com/search/history/results.uri?origin=searchhistory&shid=5) |
| 4 | ( ( TITLE-ABS-KEY ( "Arthritis, Rheumatoid" )  OR  TITLE-ABS-KEY ( "Rheumatoid Arthritis" )  OR  TITLE-ABS-KEY ( "Rheumatoid" ) ) )  AND  ( ( TITLE-ABS-KEY ( "Osteoporosis" )  OR  TITLE-ABS-KEY ( "Osteoporo*" )  OR  TITLE-ABS-KEY ( "Bone Loss" )  OR  TITLE-ABS-KEY ( "Osteopenia" )  OR  TITLE-ABS-KEY ( "Bone Density" )  OR  TITLE-ABS-KEY ( "Bone Mineral Density" ) ) )  AND  ( ( TITLE-ABS-KEY ( "Prevalence" )  OR  TITLE-ABS-KEY ( "Incidence" )  OR  TITLE-ABS-KEY ( "Epidemiology" )  OR  TITLE-ABS-KEY ( "Frequency" ) ) ) View Less | [1,594 document results](https://www.scopus.com/search/history/results.uri?origin=searchhistory&shid=4) |
| 3 | ( TITLE-ABS-KEY ( "Prevalence" )  OR  TITLE-ABS-KEY ( "Incidence" )  OR  TITLE-ABS-KEY ( "Epidemiology" )  OR  TITLE-ABS-KEY ( "Frequency" ) ) | [5,913,928 document results](https://www.scopus.com/search/history/results.uri?origin=searchhistory&shid=3) |
| 2 | ( TITLE-ABS-KEY ( "Osteoporosis" )  OR  TITLE-ABS-KEY ( "Osteoporo*" )  OR  TITLE-ABS-KEY ( "Bone Loss" )  OR  TITLE-ABS-KEY ( "Osteopenia" )  OR  TITLE-ABS-KEY ( "Bone Density" )  OR  TITLE-ABS-KEY ( "Bone Mineral Density" ) ) | [232,419 document results](https://www.scopus.com/search/history/results.uri?origin=searchhistory&shid=2) |
| 1 | ( TITLE-ABS-KEY ( "Arthritis, Rheumatoid" )  OR  TITLE-ABS-KEY ( "Rheumatoid Arthritis" )  OR  TITLE-ABS-KEY ( "Rheumatoid" ) ) | [217,651 document results](https://www.scopus.com/search/history/results.uri?origin=searchhistory&shid=1) |
